# Decreased CSF Transferrin in sCJD: A Potential Pre-Mortem Diagnostic Test for Prion Disorders

**DOI:** 10.1371/journal.pone.0016804

**Published:** 2011-03-09

**Authors:** Ajay Singh, ′Alim J. Beveridge, Neena Singh

**Affiliations:** 1 Department of Pathology, Case Western Reserve University, Cleveland, Ohio, United States of America; 2 Department of Organizational Behavior, Case Western Reserve University, Cleveland, Ohio, United States of America; Ohio State University, United States of America

## Abstract

Sporadic Creutzfeldt-Jakob-disease (sCJD) is a fatal neurodegenerative condition that escapes detection until autopsy. Recently, brain iron dyshomeostasis accompanied by increased transferrin (Tf) was reported in sCJD cases. The consequence of this abnormality on cerebrospinal-fluid (CSF) levels of Tf is uncertain. We evaluated the accuracy of CSF Tf, a ‘new’ biomarker, as a pre-mortem diagnostic test for sCJD when used alone or in combination with the ‘current’ biomarker total-tau (T-tau). Levels of total-Tf (T-Tf), isoforms of Tf (Tf-1 and Tf-β2), and iron saturation of Tf were quantified in CSF collected 0.3–36 months before death (duration) from 99 autopsy confirmed sCJD (CJD+) and 75 confirmed cases of dementia of non-CJD origin (CJD-). Diagnostic accuracy was estimated by non-parametric tests, logistic regression, and receiver operating characteristic (ROC) analysis. Area under the ROC curve (AUC), sensitivity, specificity, positive and negative predictive values (PV), and likelihood ratios (LR) of each biomarker and biomarker combination were calculated. We report that relative to CJD-, CJD+ cases had lower median CSF T-Tf (125,7093 vs. 217,7893) and higher T-tau (11530 vs. 1266) values. AUC was 0.90 (95% confidence interval (CI), 0.85–0.94) for T-Tf, and 0.93 (95% CI, 0.89–0.97) for T-Tf combined with T-tau. With cut-offs defined to achieve a sensitivity of ∼85%, T-Tf identified CJD+ cases with a specificity of 71.6% (95% CI, 59.1–81.7), positive LR of 3.0 (95% CI, 2.1–4.5), negative LR of 0.2 (95% CI, 0.1–0.3), and accuracy of 80.1%. The effect of patient age and duration was insignificant. T-Tf combined with T-tau identified CJD+ with improved specificity of 87.5% (95%CI, 76.3–94.1), positive LR of 6.8 (95% CI, 3.5–13.1), negative LR of 0.2 (95% CI, 0.1–0.3), positive-PV of 91.0%, negative-PV of 80.0%, and accuracy of 86.2%. Thus, CSF T-Tf, a new biomarker, when combined with the current biomarker T-tau, is a reliable pre-mortem diagnostic test for sCJD.

## Introduction

Sporadic Creutzfeldt-Jakob disease is a uniformly fatal prion disorder of humans that results from refolding of the normal prion protein (PrP^C^) to a β-sheet rich pathogenic conformation called PrP-scrapie (PrP^Sc^) [1], [2]. Currently, the only definitive diagnostic test for sCJD is based on detection of PrP^Sc^ in the brain tissue obtained by biopsy or at autopsy [2]. Available pre-mortem diagnostic tests have provided inconsistent results, making it difficult to use therapeutic options where available [3]–[8]. Moreover, undiagnosed cases of sCJD are likely to transmit the disease to healthy individuals, raising significant public health concerns [2], [9].

Consequently, significant effort has gone into the identification of sensitive and specific biomarkers for sCJD [8], [10], [11]. Of these, analysis of CSF proteins has proved promising due to its reasonable reliability and low cost. Commonly tested CSF proteins include 14-3-3, total tau (T-tau), S100B, and neuron-specific enolase (NSE) [12]–[17]. Several large scale studies, some across centers, have explored the accuracy of these biomarkers in the context of variables such as clinical stage of disease, valine/methionine polymorphism in the prion protein gene, and sub-type of sCJD [18]–[23]. Based on these and other considerations, the World Health Organization approved a combination of CSF 14-3-3 and EEG for the pre-mortem diagnosis of clinically suspected cases of sCJD [12], [23], [24]. Subsequent studies reported high sensitivity but relatively low specificity of this test, resulting in its replacement by a combination of CSF 14-3-3 and T-tau for cases of dementia suspected of sCJD [3]–[6], [14], [17], [25]. However, subsequent reports indicated varying sensitivity and specificity for 14-3-3 and T-tau when used singly or in combination, re-kindling the debate and dissent concerning the diagnostic accuracy of this test [3]–[6], [14], [17], [27]–[31].

Major factors contributing to the inconsistent performance of 14-3-3 and T-tau combination include: 1) variable false positives since 14-3-3 and T-tau are elevated in several other dementias besides sCJD [29]–[31], 2) experimental variability of 14-3-3 that is semi-quantitative at best [4]–[6], and 3) lack of comparative analysis of biomarker accuracy within and across studies using a rigorous statistical approach [12]–[23]. To address these weaknesses, it is critical to identify a biomarker that is altered by sCJD associated pathology and therefore specific, abundant in the CSF and easily quantifiable, and superior in diagnostic accuracy relative to the current biomarkers 14-3-3 and T-tau using uniform benchmarks of analysis.

One such potential biomarker is Tf that is increased in sCJD and prion disease affected animal brains [32]–[36]. The effect of this change on CSF levels of Tf, however, is not known. In this study, we analyzed levels of CSF Tf in 99 autopsy confirmed cases of sCJD (CJD+) and 75 cases of dementia of non-CJD origin (CJD-) to evaluate the potential of CSF Tf and its isoforms Tf-1 and Tf-β2 as ‘new’ diagnostic biomarkers of sCJD. The diagnostic accuracy of new biomarkers was compared with the ‘current’ biomarker T-tau using common standards of evaluation and rigorous statistical modeling. Finally, the diagnostic accuracy of optimal ‘new’ biomarker and T-tau combination was compared with the 14-3-3 and T-tau combination currently used for the pre-mortem diagnosis of sCJD.

## Methods

### Ethics statement

Human tissue and CSF samples were obtained from the National Prion Disease Pathology Surveillance Center (NPDPSC) at Case Western Reserve University, Ohio. All samples are from deceased subjects, and personal information is limited to age, sex, symptoms, neuropathology, and classification of prion disease. The use of these samples has been granted waiver of informed consent by the Case Western Reserve University Institutional Review Board since the protocol meets criteria for exemption under Federal regulations 45 CFR 46.101 (b).

#### Human samples

To avoid sample bias, pre-mortem CSF from autopsy-confirmed cases of sCJD (CJD+) and dementia of non-CJD origin (CJD-) received between 2006 and 2008 were included in this study. The samples consisted of 99 CJD+ and 75 CJD- cases, amounting to a total of 174 cases. Specific diagnosis of CJD- cases included Alzheimer's Disease (AD) (17), encephalitis (3), meningoencephalitis (2), cortical dysplasia (1), cortical angiopathy (1), angiitis with microinfarcts (1), infarct (1), cerebral vasculopathy (1), Lewy body dementia (1), Lewy body variant of Alzheimer's disease (1), anoxic and Wernicke's encephalopathies (1), meningeal carcinomatosis (1), Alexander disease (1), leukoencephalopathy (1), hippocampal sclerosis (1), frontotemporal dementia (1), coccidioidomycosis meningoencephalitis (1), CNS lymphoma (1), chronic meningoencephalitis (1), epilepsy or encephalopathy (1), glioma (1), perivenous encephalomyelitis (1), CNS lymphoma (1), military metastases (1), leptomeningeal lymphomatosis/leukemia (1), mitochondrial encephalopathy (1), lymphoma (1), and non-CJD dementia of uncertain diagnosis (29). The age at autopsy ranged from 37–85 years for CJD+, and 47–84 years for CJD- cases. All CSF samples were stored at −80°C until use. Human brain tissue from the frontal cortex of CJD+ and age-matched cases of dementia (CJD−) was obtained from the NPDPSC and stored at −80°C (brain tissue and CSF samples are from different cases). Tissue homogenates (10%) were prepared in lysis buffer and analyzed by Western blotting following standard protocols.

#### Western blot and ELISA

Equal volume of CSF from CJD+ and CJD- cases was fractionated by SDS-PAGE and proteins transferred to PVDF membranes were probed with anti-transferrin antibody (Genetex Inc., Cat # GTX 21223) followed by HRP-conjugated secondary antibody and visualization of reactive bands with ECL (Amersham). Several procedural and statistical precautions were taken to reduce error in comparing multiple samples. Procedurally, a similar protocol was followed for all Western blots, including exposure times. In addition, strips of PVDF membrane representing specific molecular weights were exposed to X-ray film simultaneously in large cassettes to obtain similar exposure. Quantification of immunoreactive bands was performed with UN-SCAN-IT software (version 6.1, Silk Scientific Inc., Utah, USA) using three exposures from a single membrane showing exponential increase in intensity. Statistically, results from different Western blots were analyzed simultaneously in logistic regression by treating these as clustered observations. The statistical software Stata allows estimation of regression coefficients after controlling for clustering to produce unbiased standard errors. Two Stata procedures were used to do this: the ‘vce cluster’ option and svy option. Each logistic regression model was first tested without adjustment for clustering and then tested with each of the 2 clustering options. Both had the effect, in general, of increasing standard errors and p-values for the coefficient estimates as expected. However, the significance of the coefficients of the biomarkers did not change; all their coefficients remained significant at the p<.05 level. T-Tf (ELISA) was estimated with Human Transferrin ELISA kit (Alpha diagnostic international Inc., Cat # 1210) following the manufacturer's instructions. T-tau was determined by Human Tau (total) ELISA Kit (Invitrogen, Camarillo, CA; KBH0042) as directed by the manufacturer. Iron saturation of CSF Tf was determined by radiolabeling with ^59^Fe followed by fractionation on native gradient gels and autoradiography.

### Statistical analysis

The following new biomarkers were evaluated for their diagnostic accuracy: total transferrin by Western blot (T-Tf (WB)) and ELISA (T-Tf (ELISA)) techniques, Tf-1, Tf-β2, and iron saturation of Tf (^59^Fe-Tf). Of these, T-Tf, Tf-1, and Tf-β2 offered greater promise, and were analyzed further either alone or in combination with T-tau, a current biomarker that is quantifiable and is superior to other CSF biomarkers used for the diagnosis of sCJD [14], [17], [21], [28].

A combination of univariate and multivariable statistical methods were used to evaluate the relative diagnostic accuracy of individual biomarkers. Since the biomarkers were expected to evidence skewed distributions, nonparametric statistical procedures were used as these are subject to fewer distributional assumptions and provide less biased estimators when data are non-normal. For relative analysis of individual biomarkers, the nonparametric Mann-Whitney *U* test was used for focused comparisons to test if the levels of new and current biomarkers differ significantly between CJD+ and CJD-, and between CJD+ and AD cases. In addition, correlation analysis was performed using Spearman's rank correlation (ρ), which is also nonparametric. Finally, logistic regression, which makes no assumptions regarding the distributional properties of data (e.g., non-normal) [37] was used to parameterize the diagnostic accuracy of biomarkers after controlling for age and duration (interval between CSF collection and death). Because the data were clustered (four sets of Western blots), logistic regression was conducted in Stata 10 with the option to correct standard errors for clustering. This option is known to correct for underestimates of coefficient standard errors, resulting in more conservative results.

Using logistic regression results, an analytic expression for the risk of CJD was derived for each individual biomarker. Area under the receiver operating characteristic (ROC) curve and the Aikake Information Criterion (AIC) were obtained. A ROC curve graphically shows the trade-offs between sensitivity and specificity for different cut-offs used to discriminate between positive and negative cases (i.e., CJD+ and CJD- cases). The area under the ROC curve (AUC) can be understood as an estimate of the probability that the biomarker being tested correctly ranks a CJD+ case higher than a CJD- case and, therefore, indexes the discriminating power of the biomarker. The AIC is used to compare different logistic regression models for different biomarkers (individually or in combination). A model with a relatively lower AIC is considered superior due to its better fit to data and its parsimony. For each model, estimates of specificity and positive and negative likelihood ratios (LR) were obtained given a baseline sensitivity of 85%, which has precedence in the literature as a reasonable cut-off level for biomarker comparison of Alzheimer's disease (AD) [38], [39]. Positive LR is defined as sensitivity/(1-specificity) and represents the increase in likelihood of a positive test result if the disease is present compared to its being absent. The negative LR is defined as (1-sensitivity)/specificity and represents the decrease in likelihood of a negative test result if the disease is present compared to it being absent. Positive and negative LR, like specificity, depends on the chosen cut-off for sensitivity (set at 85% in this study). Confidence intervals for proportions were calculated according to the efficient-score method and corrected for continuity [40]. Confidence intervals for LRs were calculated as proposed in a previous report [41]. All statistical tests were conducted at the 95% confidence level.

To identify an optimal combination of biomarkers, all two way combinations of new and current biomarkers were also entered into a logistic regression model with controls for age, duration, and clustered data (as above). Combining new and current biomarker T-tau was intended to take advantage of the differential pathogenic mechanisms represented by these biomarkers. We focused on two-way combinations for reasons of practicality. However, a specific combination involving T-Tf and 14-3-3 could not be evaluated because the readouts for 14-3-3 are not precisely quantifiable, and this biomarker has demonstrated relatively low specificity in previous studies [4]–[6].

All models were compared using three criteria: AUC, specificity, and AIC [42]. Model comparisons were based on multiple indicators while recognizing that specificity at a given level of sensitivity is a key discriminating factor. Models with higher specificity were preferred. The optimal biomarker or combination of biomarkers was expected to ideally have the highest AUC and specificity, and the lowest AIC. We also conducted power analysis as proposed in a previous report [43]. We determined that the new biomarkers would possess a discriminating power at least as good as T-tau; thus we based our power calculation on the expected effect size of T-tau. From previously published data on the power of T-Tau to discriminate between CJD+ cases and other forms of dementia [44]–[46], we calculated an expected standardized difference of at least 1.7. Based on this, our study yields an estimate of statistical power exceeding 0.99 for logistic regression as well as the Mann-Whitney *U* test.

## Results

### Tf levels are decreased in the CSF of sCJD cases

**Figure 1:**
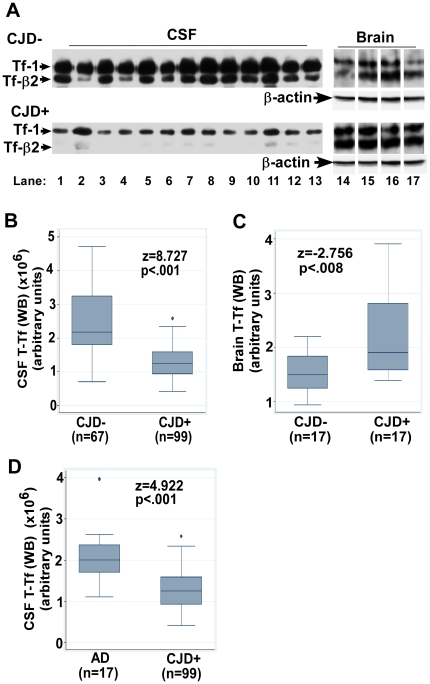
Tf is decreased in the CSF and increased in the brain of CJD+ cases. (**A**) Immunoblotting for Tf shows reduced levels of Tf-1 and Tf-β2 in the CSF of CJD+ relative to CJD- samples (lanes 1–13), and increased levels of both isoforms in the brain tissue of CJD+ relative to CJD- samples (lanes 14–17) (55). (**B**–**D**) Box and whisker plots of T-Tf (WB) indicating the median, 25–75^th^ percentiles, and outliers. The top and bottom of the “box” represent 75^th^ and 25^th^ percentile respectively. The “whiskers” represent the highest and lowest values. Outliers are values over 1.5 times the interquartile range and are shown as circles away from the whiskers. Significance of biomarker differences between CJD- vs. CJD+ and AD vs. CJD+ was calculated by the M-W test.

Evaluation of CSF Tf by Western blot (WB) reveals two distinct bands representing Tf-1 and Tf-β2, the latter representing deglycosylated Tf specific to the brain and CSF (Figure 1 A, lanes 1–13). Total Tf (T-Tf (WB)) comprising of Tf-1 and Tf-β2 collectively, and individual subunits i.e. Tf-1 and Tf-β2 individually are decreased in the CSF of CJD+ relative to CJD- cases (Figure 1 A, lanes 1–13). In contrast, brain homogenates show a relative increase in T-Tf (WB), Tf-1, and Tf-β2 in CJD+ relative to CJD- cases (Figure 1 A, lanes 14–17) (cut lanes were re-aligned for comparison. Original blot is shown in Figure S1).

**Figure 2:**
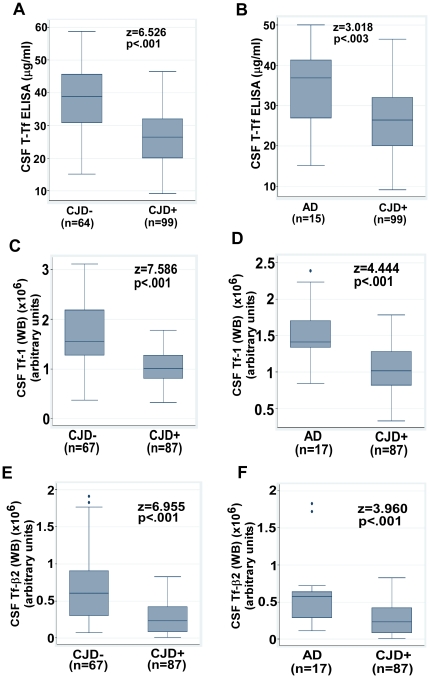
Quantitative comparison of CSF Tf between CJD+ and other dementias by the M-W *U* test. (**A–F**) Box and whisker plots of different biomarkers indicating the median, 25–75^th^ percentiles, and outliers as in Figure 1 B–D. Significance of biomarker differences between CJD- vs. CJD+ and AD vs. CJD+ was calculated by the M–W test.

Quantitative comparison shows a decrease in CSF T-Tf (WB) by 49% (z = 8.73, p<.001) and an increase in brain T-Tf (WB) by 39% (z = 2.76, p<.008) in CJD+ relative to CJD- cases (Figure 1 B, C). Comparison with AD cases shows a decrease in CSF T-Tf (WB) by 39% in CJD+ relative to AD cases (z = 4.92, p<.001) (Figure 1 D). Estimation of CSF T-Tf by ELISA shows a decrease of 30% in CJD+ relative to CJD- (z = 6.53, p<.001), and 26% relative to AD cases (z = 3.02, p  = .003) (Figure 2 A, B). The difference in T-Tf measured by WB and ELISA methods is due to optimization of the ELISA kit for serum Tf that lacks Tf-β2, and is therefore less accurate in estimating brain T-Tf.

Comparison of individual subunits of Tf shows a decrease in CSF Tf-1 by 35% (z = 7.59, p<.001) and Tf-β2 by 61% (z = 6.96, p<.001) in CJD+ relative to CJD- samples (Figure 2 C, E), and a decrease of 28% in Tf-1 (z = 4.44, p<.001) and 59% in Tf-β2 (z = 3.96, p<.001) relative to AD samples (Figure 2 D, F).

To compare the iron content of CSF Tf between CJD- and CJD+ cases, first the iron saturation of CSF Tf from a relatively normal CJD- case was determined by competing radiolabeled ^59^FeCl_3_ with decreasing concentrations of unlabeled FeCl_3_ and fractionating ^59^Fe-Tf on a native gel for quantification (Figure S2 A, B). These results demonstrate that CSF Tf is not fully saturated with iron (Figure S2 B). Subsequently, CSF samples representing equal concentration of Tf (determined by ELISA) from CJD- and CJD+ cases were incubated with equal counts of ^59^FeCl_3_ to radiolabel Tf, dialyzed to remove free ^59^FeCl_3_, and fractionated on a native gel (Figure S2 C). Quantification of ^59^Fe-Tf shows equivalent saturation of CSF Tf from CJD-, CJD+, and AD samples, indicating normal iron binding capacity CSF Tf regardless of the underlying disease (Figure S2 D, E).

### Biomarker levels and differential diagnostics of T-Tf as a new biomarker for sCJD

**Figure 3:**
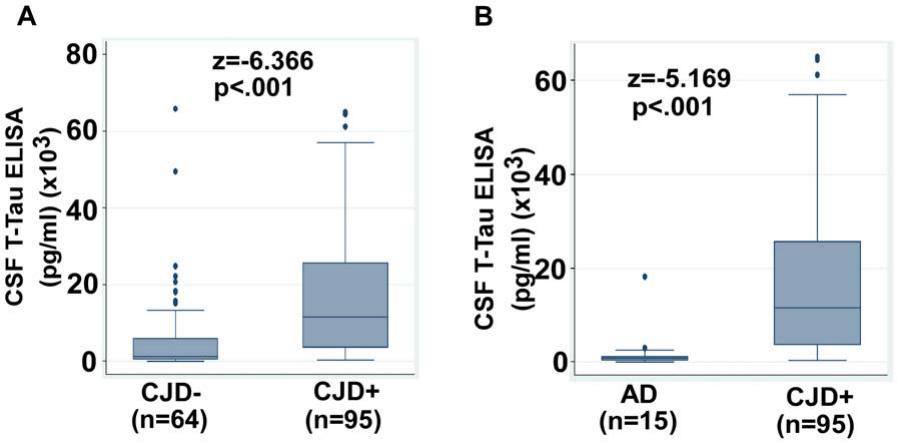
Quantitative comparison of CSF T-tau between CJD+ and other dementias by the M–W *U* test. (**A & B**) Box and whisker plots of T-tau comparing CJD- vs. CJD+ and AD vs. CJD+ cases. Plot shows median, 25 to 75^th^ percentiles, and outliers as in Figures 1 and 2.

Comparison of different biomarkers using Mann-Whitney *U* test reveals that the new biomarkers T-Tf (WB), Tf-1, and Tf-β2 are more sensitive differentiators of the CJD+ vs. CJD- groups in comparison to the current biomarker T-tau (Table 1, Figures 2 A–F and 3 A, B). Relative to T-tau, the absolute z-values for T-Tf (WB) and Tf-1 are higher by at least 2 and 1 standard deviation (SD) respectively (Table 1). Noting that differential diagnosis is meaningful if the biomarker discrimination exceeds at least 2 SD, the current use of T-tau biomarker provides equivalent diagnostic accuracy as Tf-β2 (absolute z = 6.37 and 6.96 respectively) and Tf-1 (Δz = 1.22), but significantly poorer than T-Tf (WB) (Δz = 2.36) (Table 1, Figures 2 A, C, E and 3 A). However, the diagnostic accuracy of new biomarkers in differentiating CJD+ from AD does not yield comparable superiority over T-tau. Rather, these biomarkers evidence similar discrimination. T-tau has the highest z-value (z = −5.17, p<.001), but its diagnostic accuracy is equivalent to T-Tf, Tf-1 and Tf-β2 (Δz = 0.25, 0.73 and 1.21 respectively) (Table 1, Figure 1 D, Figures 2 B, D, F and 3 B). (In hypothesis testing, at a confidence level of 95%, any difference in z-values greater than 1.96 is significant. Based on this we reasoned that when z-values corresponding to individual biomarkers differ by 2 or more, the biomarker with greater z-value provides significantly greater discrimination at p = .05).

**Table 1:** Individual biomarker performance by the Mann-Whitney *U* test.

Comparison test	New Biomarkers				Current Biomarker
	T-Tf (WB)	Tf-1	Tf-β2	T-Tf (ELISA) (µg/ml)	T-tau (pg/ml)
**CJD+ vs. CJD-**					
CJD+ median (n)	1257093 (99)	1017031 (87)	236243 (87)	26.46 (99)	11530.87 (95)
CJD- median (n)	2177893 (67)	1555881 (67)	606359 (67)	38.90 (64)	1266.97 (64)
M–W U Test					
* Z-statistic*	8.73	7.59	6.96	6.53	−6.37
* p-value*	<.001	<.001	<.001	<.001	<.001
**CJD+ vs. AD only**					
CJD+ median (n)	1257093 (99)	1017031 (87)	236243 (87)	26.46 (99)	11530.87 (95)
AD median (n)	2009259 (17)	1413527 (17)	577539 (17)	36.89 (15)	876.40 (15)
M–W U Test					
* Z-statistic*	4.92	4.44	3.96	3.02	−5.17
* p-value*	<.001	<.001	<.001	0.003	<.001

Note: The n ranges from 87 to 99 for biomarkers in the CJD+ group and from 64 to 67 for the CJD- group due to insufficient CSF volume. To ensure that varying n does not introduce bias, an additional analysis was performed using cases with only non-missing values for all biomarkers (87 for CJD+ and 64 for CJD-). The results did not differ noticeably from those reported above.

After controlling for patient age and duration, T-tau showed significant correlation with duration in CJD+ (ρ = −0.35, p<.001), but not in CJD- cases (ρ = −0.14, p = 0.28). None of the new biomarkers showed a significant correlation with duration in the CJD+ or CJD- group. (Correlation of T-Tf (WB), Tf-1, and Tf-β2 in CJD+ group ρ = −0.08, p = 0.41; ρ = −0.02, p = 0.89; ρ = 0.11, p = 0.29, respectively, and in CJD- group ρ = 0.06, p = 0.62; ρ = 0.01, p = 0.96; ρ = 0.06, p = 0.64, respectively). These results indicate that T-tau changes as sCJD progresses, while the new biomarkers remain fairly stable in CJD+ and CJD- cases. None of the biomarkers showed any correlation with age.

For comparative analysis of new and the current biomarker T-tau, logistic regression analysis was performed. Only T-Tf (WB), not T-Tf (ELISA) results are considered for reasons mentioned above. When individual biomarkers are tested for their diagnostic accuracy, the new biomarkers T-Tf (WB), Tf-1, and Tf-β2 are superior to the current biomarker T-tau (Table 2). T-Tf (WB) and Tf-1 have significantly higher AUC (0.90 and 0.86 respectively) than T-Tau (0.78). Tf-β2 also has a higher AUC (0.82) than T-tau, but the difference is not significant. T-Tf (WB), Tf-1, and Tf-β2 yield significantly higher specificities of 71.6%, 65.7% and 64.2% respectively, while T-tau shows a relatively low specificity of 48.4% (see discussion below). Finally, the AICs for T-Tf (WB), Tf-1 and Tf-β2 of 0.81, 0.96 and 1.03 respectively are all lower than the AIC for T-Tau (1.23) (Table 2).

**Table 2:** Comparison of ‘new’ and ‘current’ biomarkers by logistic regression.

Statistic	New Biomarkers (n)				Current biomarker (n)	New + Current
	T-Tf (WB) (99)	Tf-1 (87)	Tf-β2 (87)	T-Tf ELISA (99)	T-tau (95)	T-Tf (WB)+T-tau
Area under ROC (95%CI)	0.90 (0.85–0.94)	0.86 (0.80–0.92)	0.82 (0.76–0.89)	0.80 (0.73–0.88)	0.78 (0.71–0.85)	0.93 (0.89–0.97)
Sensitivity (95%CI)	85.9 (77.1–91.8)	85.1 (75.4–91.5)	85.1 (75.4–91.5)	85.9 (77.1–91.8)	85.3 (76.2–91.4)	85.3 (76.2–91.4)
Specificity (95%CI)	71.6 (59.1–81.7)	65.7 (53.0–76.6)	64.2 (51.5–75.3)	64.1 (51.0–75.4)	48.4 (35.9–61.2)	87.5 (76.3–94.1)
Positive LR (95%CI)	3.0 (2.1–4.5)	2.5 (1.8–3.5)	2.4 (1.7–3.3)	2.4 (1.7–3.3)	1.7 (1.3–2.1)	6.8 (3.5–13.1)
Negative LR (95%CI)	0.2 (0.1–0.3)	0.2 (0.1–0.4)	0.2 (0.1–0.4)	0.2 (0.1–0.4)	0.3 (0.2–0.5)	0.2 (0.1–0.3)
PPV (%) (95%CI)	81.7 (72.7–88.4)	76.3 (66.4–84.1)	75.5 (65.6–83.4)	78.7 (69.6–85.8)	71.1 (61.7–79.0)	91.0 (82.6–95.8)
NPV (%) (95%CI)	77.4 (64.7–86.7)	77.2 (63.8–86.8)	76.8 (63.3–86.6)	74.5 (60.7–84.9)	68.9 (53.2–81.4)	80.0 (68.4–88.3)
AIC	0.81	0.96	1.03	1.06	1.23	0.70
Accuracy	80.1	76.6	76	77.3	70.4	86.2

Note: Accuracy was defined as true positives + true negatives/total number tested.

Cut-off was chosen to achieve a sensitivity of ∼85% [38], [39].

Positive/negative LR results are robust to variations in prevalence rates between the study and overall population, and are provided in addition to PPV/NPV that are highly dependent on the population tested.

Among the new group of biomarkers, T-Tf (WB) has by far the highest diagnostic accuracy relative to other individual biomarkers. Thus, T-Tf (WB) yields an AUC of 0.90 that is significantly higher than corresponding values for other biomarkers with the exception of Tf-1. The AIC associated with the logistic regression model using T-Tf (WB) is lowest across all other models. Finally, the specificity of T-Tf (WB) is significantly higher than that of T-Tau and equivalent to that of Tf-1 and Tf-β2. However, T-Tf (ELISA) does not have the same superior characteristics: the model has a lower AUC (0.81), higher AIC (1.06), and poorer specificity of 64.1% for reasons mentioned above.

### Optimal prediction of sCJD

**Figure 4:**
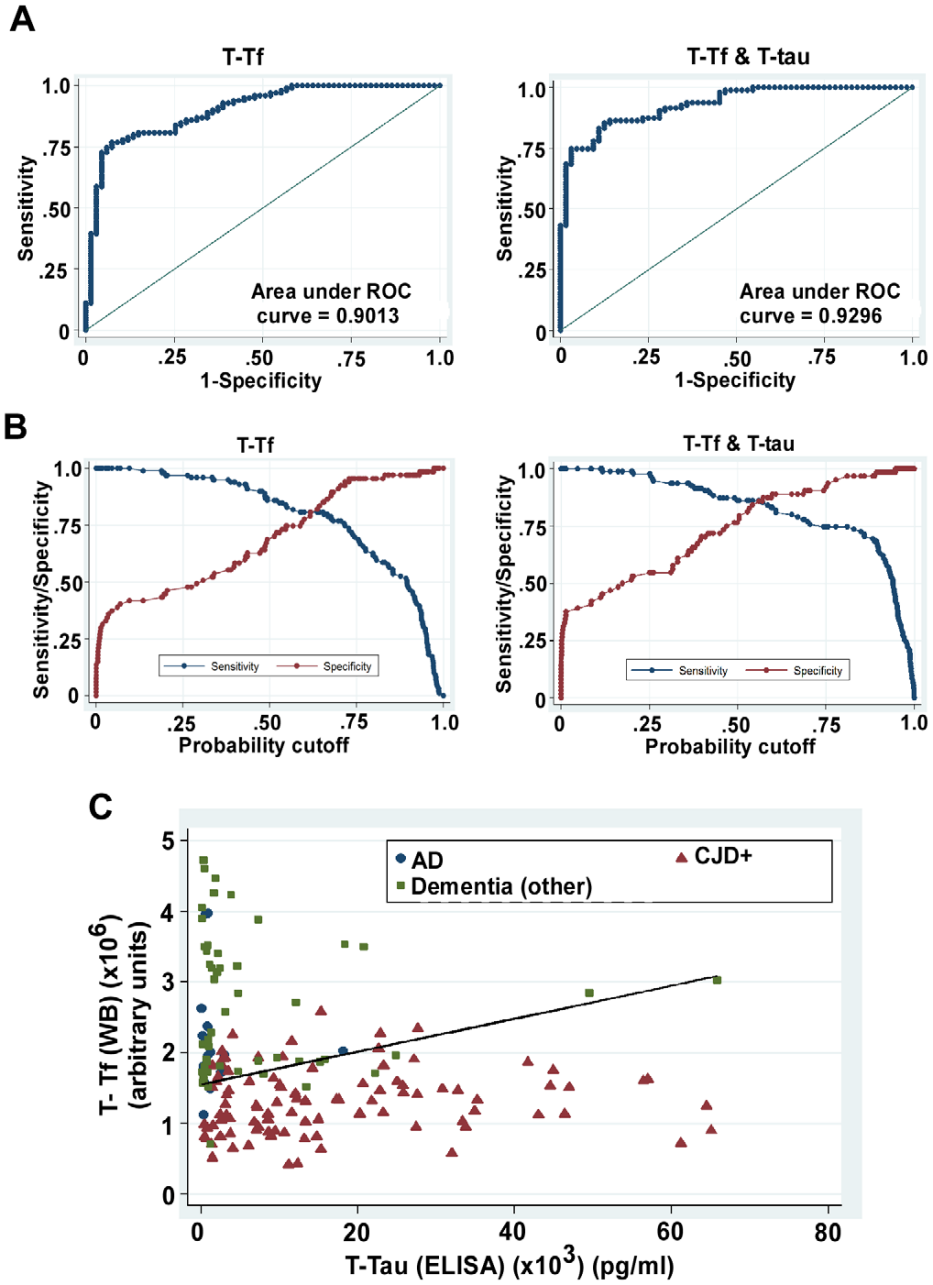
Potential of CSF T-Tf alone or in combination with T-tau as a diagnostic test for CJD+. (**A & B**) ROC curve of T-Tf (WB) and T-Tf (WB) in combination with T-tau. (**C**) Scatter graph shows separation of CJD+ from cases of AD and other dementias. Reference line shows the cut-off equation derived to achieve a sensitivity of ∼85%.

Past research shows that relative to any single biomarker, using two biomarkers in combination generally enhances the prediction of individuals likely to suffer from AD or sCJD [26], [39]. Accordingly, different combinations of new biomarkers and T-tau were evaluated for their diagnostic accuracy. An optimal predictive model is achieved when T-Tf (WB) is used in conjunction with T-Tau (Figure 4). This combination yields an AUC of 0.93 (highest of all combinations except for T-Tf (WB) where the difference is insignificant), specificity of 87.5% (highest of all combinations), and AIC of 0.7 (lowest of all combinations) (Figure 4, panel A, Table 2). The addition of a second predictor variable makes the model less parsimonious compared to models including only a single biomarker, which normally, all else being equal, increases the AIC. The fact that this model has the lowest AIC is therefore remarkable; it indicates that the gain in fit to the data compared to other models more than makes up for the decrease in parsimony. The superiority of this combination over individual biomarkers is also clear in its positive LR (6.8) which is significantly higher than all other biomarkers, positive predictive value of 91%, and higher accuracy of 86.2% relative to each biomarker individually (Table 2, Figure 4). Finally, an equation was constructed based on logistic regression analysis for the combination of T-Tf (WB) and T-tau such that the sensitivity for CJD+ would approximate 85% [38], [39]. To visualize the discriminative power of T-Tf (WB) (y) and T-tau (x) combination, this equation was plotted as a line in the scatter plot of CJD, AD and other dementia cases (Figure 4, panel C).

## Discussion

This report describes the accuracy of new biomarkers CSF T-Tf (WB) including its two isoforms Tf-1 and Tf-β2 in identifying CJD+ from dementia of other causes. Using rigorous statistical modeling and analysis of new and currently used biomarker T-tau, we demonstrate that each of the new biomarkers is superior to T-tau, and the combination of T-Tf (WB) and T-tau is more accurate in identifying sCJD than the currently used combination of 14-3-3 and T-tau. Below, we discuss distinctive features of the methodology used for comparing new and current biomarkers, possible reasons for high specificity of new biomarkers when used singly and significant improvement in diagnostic accuracy when combined with T-tau, and limitations of our study leading to directed avenues in the identification of accurate CSF biomarker(s) for sCJD.

A rigorous statistical approach was used to examine the significance (nonparametric M–W test), quantitative comparison using uniform criteria (logistic regression), and sensitivity-specificity interdependence (ROC) of different biomarkers. In contrast to specific cut-off values for individual biomarkers, a preset sensitivity of 85% was applied to all biomarkers to provide a common quantitative baseline for comparison [38], [39]. Though this method has been used in the past for evaluation of biomarkers of AD [39], to our knowledge, this is the first report where a similar uniform approach has been applied to compare different biomarkers of sCJD. By this approach, the new biomarkers T-Tf (WB) and its isoforms Tf-1 and Tf-β2 performed better than the current biomarker T-tau in all parameters tested, i.e. sensitivity, specificity, AUC, AIC, and accuracy. Comparison among the new biomarkers revealed that T-Tf (WB) is superior to Tf-1 and Tf-β2 in all of the above parameters. This is probably because the ratio of Tf-1 and Tf-β2 varies between different cases, and the sum of these two isoforms represented by T-Tf provides a more accurate estimate of CSF Tf levels. The relative superiority of new biomarkers over T-tau is probably due to their association with sCJD associated pathology, in particular imbalance of brain iron homeostasis that has emerged as a common feature of sCJD and prion disease affected experimental models [32]–[36]. Diseased brains show a phenotype of iron deficiency in the presence of increased brain iron, suggesting sequestration of iron in biologically unavailable complexes [32]. Reflection of this abnormality as altered levels of CSF Tf therefore provides a disease associated biomarker, a significant improvement over the surrogate biomarkers currently used for the diagnosis of sCJD [3]–[8], [11]–[18].

However, instead of the compensatory increase in CSF Tf in response to brain iron deficiency as observed in cases of Restless Leg Syndrome [47]–[49], CSF Tf is significantly reduced in sCJD cases. A simplistic explanation for this observation is increased uptake by the iron starved brain tissue, reducing levels of Tf in the brain interstitial fluid and CSF that form a common compartment. However, this is difficult to comprehend given the high metabolic demand of brain cells for iron and the consequent tight regulation of brain iron metabolism [50], [51]. It is likely that accumulated iron in PrP^Sc^-protein complexes mitigates the signal for iron deficiency, reducing Tf secretion [32], [50], [51]. We did not note a significant difference in iron saturation of Tf between the two groups, ruling out dysfunction of brain Tf as the underlying cause of brain iron deficiency. It is surprising that age and duration had insignificant effects on CSF T-Tf levels. This result is counter-intuitive given the increase in brain Tf with disease progression [32], [52]. Our inability to detect a change in CSF Tf with duration may simply be due to the late stage in the incubation period when CSF samples were collected. A progressive decrease in CSF Tf may become apparent if a single patient is followed over time.

Regardless of the underlying cause, the difference in CSF Tf between CJD+ and CJD- cases is noted much before end-stage disease, providing a useful pre-mortem diagnostic biomarker for sCJD. When combined with the surrogate biomarker T-tau, the diagnostic accuracy of T-Tf (WB) and T-tau improves significantly, and is superior to the reported accuracy of 14-3-3 and T-Tau combination [16], [17], [27]. The dramatic improvement in diagnostic accuracy of T-Tf and T-tau combination is probably due to the distinct pathological processes represented by each biomarker; T-Tf representing brain iron status, and T-tau the extent of neuronal death [10]. Since brain iron deficiency is likely to induce neuronal death, the two processes may be related, altering these biomarkers in unison and therefore complementing their diagnostic capability.

Contrary to the norm, a specific cut-off value for T-Tf or T-tau was not identified to calculate the specificity and sensitivity of these biomarkers. For T-Tf we thought it pre-mature to decide on such a value due to the limited sample size. For T-tau we did not use the conventional cut-off of 1200 or 1300 pg/ml for three reasons: 1) a consistent parameter was essential for comparing T-Tf and T-tau singly or in combination, 2) majority of CJD- samples were collected in the last month before death when T-tau is likely to be released into the CSF from damaged neurons, and 3) inclusion of conditions such as AD and brain inflammation that are associated with increased levels of T-tau in the CSF [29]–[31]. The latter two conditions also explain the low specificity of T-tau observed in our study relative to published reports [17], [26], [27]. However, our samples represent a typical CJD- group presenting for diagnostic testing, and partly explain the wide variation in the specificity of T-tau reported by different centers [15], [25]. This is a major challenge since most dementias associated with brain inflammation are curable if identified early, and underscores the need for a diagnostic test of high specificity to avoid misdiagnosis as sCJD for which there is currently no treatment. It is encouraging that by combining T-Tf (WB) with T-tau the specificity increases from 71.6% for T-Tf and 48.4% for T-tau to 87.5% for the T-Tf and T-tau combination, indicating that these markers complement each other in their diagnostic capability.

In addition to its superior sensitivity and specificity, CSF Tf by itself and in combination with T-tau offers several additional advantages as a biomarker for sCJD: 1) CSF T-Tf reflects prion disease associated brain iron imbalance and is therefore likely to be more specific [32], [53], 2) significant decrease in CSF Tf is noted >12 months before end-stage disease, providing an opportunity for early diagnosis, 3) Tf is relatively resistant to limited degradation by proteinase-K [32], ensuring consistent results even in poorly preserved CSF samples, 4) since Tf-β2 displays significant discriminatory power, blood contaminated CSF samples can be used for testing, 5) levels of CSF Tf are in the µg/ml range relative to 14-3-3 and T-tau that are in the pg/ml range, allowing accurate detection from a small sample volume without the necessary step of pre-absorption of albumin and immunoglobulins, and 6) CSF T-Tf is quantifiable.

In conclusion, reduced levels of CSF Tf reinforce previous reports indicating the association of brain iron dyshomeostasis with prion disease pathology, and offer promise as a pre-mortem diagnostic test for sCJD. Although alteration of CSF Tf in sCJD cases has been described previously [53], [54], the small number of cases analyzed precludes direct comparison of these reports with our results. Nevertheless, these studies provide confidence that CSF Tf holds promise as biomarker of sCJD. Evaluation of additional CSF samples from sCJD and other forms of prion disorders and comparison with cases of rapid onset dementia will validate these observations further, and probably lead to the optimization of current automated procedures for quantifying serum Tf to CSF Tf and provide a quick and sensitive pre-mortem diagnostic test for sCJD and perhaps other human and animal prion disorders.

## Supporting Information

Figure S1 **Tf is increased in the brain tissue of CJD+ cases.** Original immunoblot of brain Tf from CJD+ and CJD- cases.
(TIF)

Figure S2 **Iron saturation of CSF Tf is comparable in CJD+ and CJD- cases.** (**A**) Competition of ^59^FeCl_3_ with graded concentrations of unlabeled FeCl_3_ for binding to CSF Tf from a CJD- case. (**B**) Standard curve demonstrating iron saturation of CSF Tf from a CJD- case. (**C**) Level of ^59^Fe-Tf in the CSF of CJD+ and CJD- cases is similar. (**D & E**) Difference between iron saturation of CSF Tf from CJD+, CJD-, and AD cases is not significant.
(TIF)
