# Supplementary figures and images for: Decreased CSF Transferrin in sCJD: A Potential Pre-Mortem Diagnostic Test for Prion Disorders
Source: PLoS One. 2011 Mar 9;6(3):e16804. doi: 10.1371/journal.pone.0016804 (PMC3052312; doi:10.1371/journal.pone.0016804)

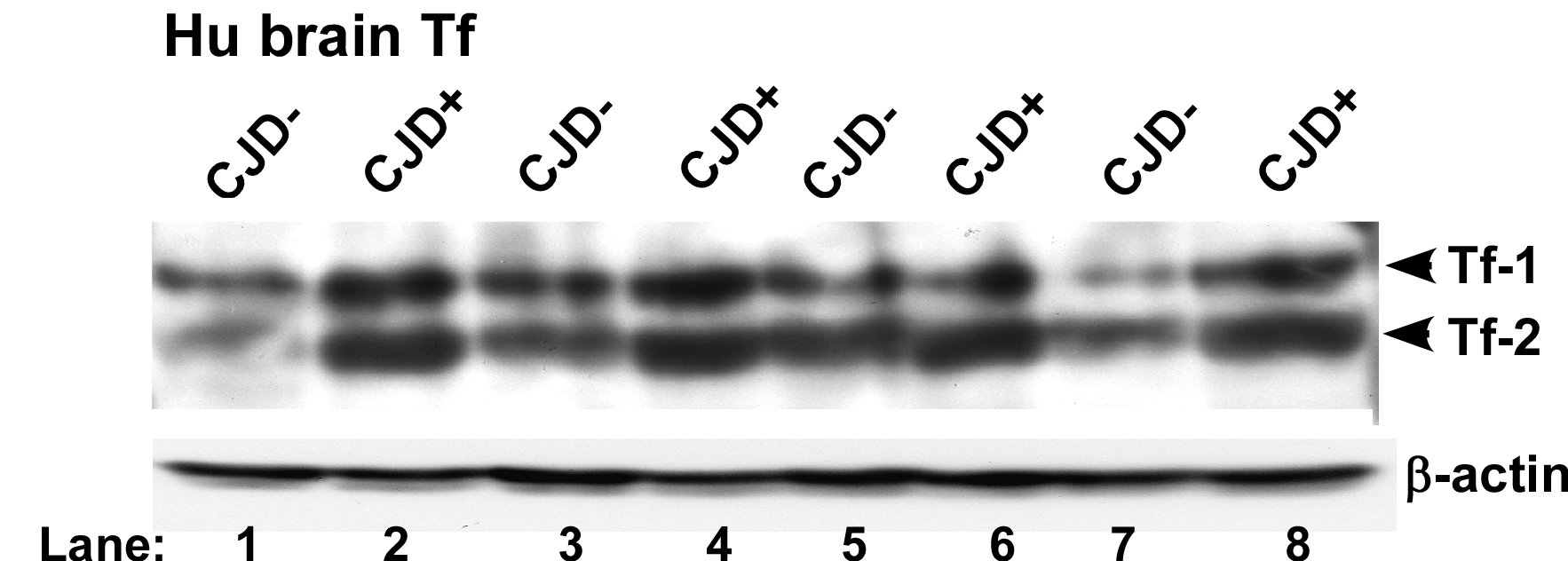

Supplement: Figure S1 — Tf is increased in the brain tissue of CJD+ cases. Original immunoblot of brain Tf from CJD+ and CJD- cases. (TIF) [file pone.0016804.s001.tif]

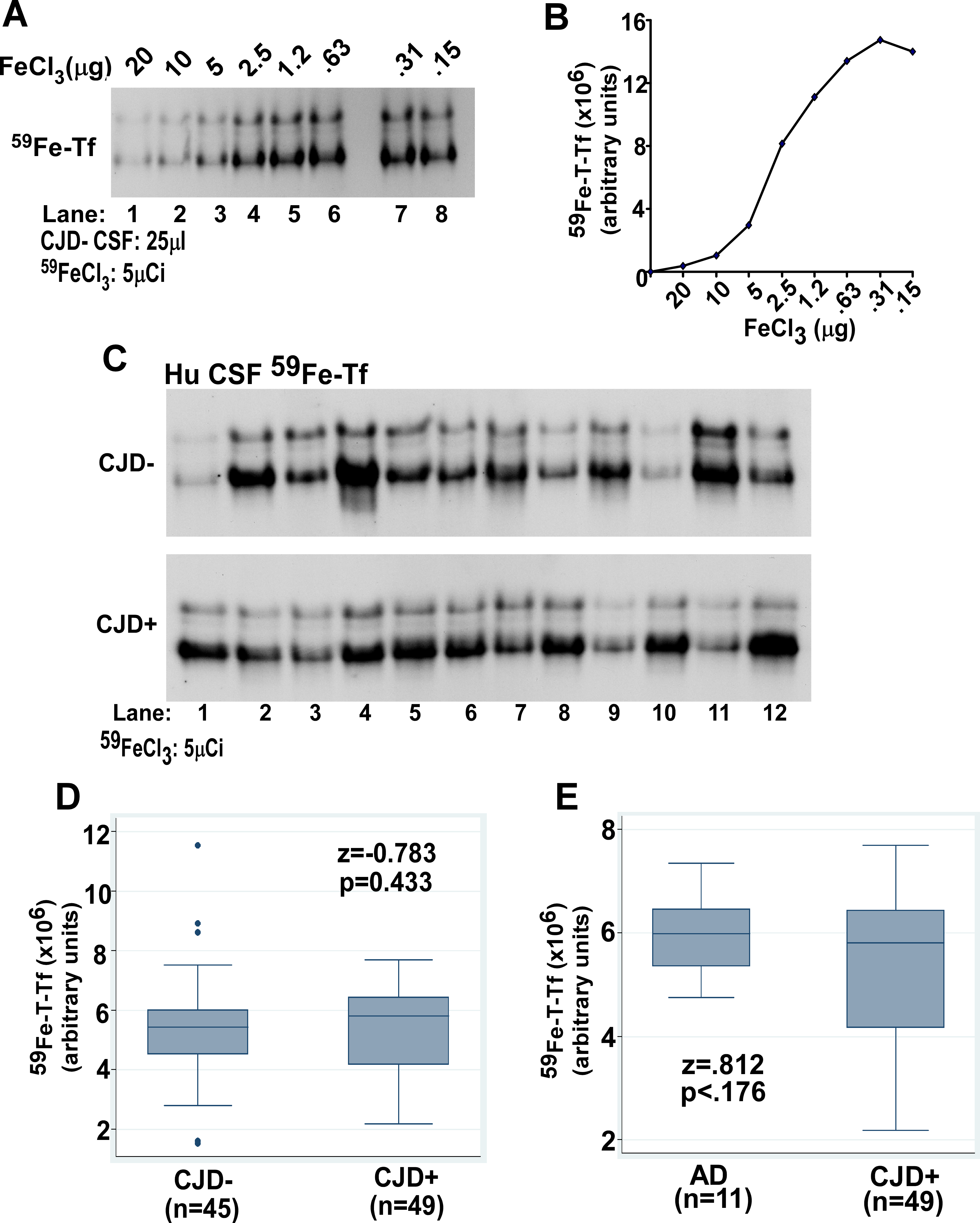

Supplement: Figure S2 — Iron saturation of CSF Tf is comparable in CJD+ and CJD- cases. (A) Competition of 59FeCl3 with graded concentrations of unlabeled FeCl3 for binding to CSF Tf from a CJD- case. (B) Standard curve demonstrating iron saturation of CSF Tf from a CJD- case. (C) Level of 59Fe-Tf in the CSF of CJD+ and CJD- cases is similar. (D & E) Difference between iron saturation of CSF Tf from CJD+, CJD-, and AD cases is not significant. (TIF) [file pone.0016804.s002.tif]
